# How to make Asthma Right Care ‘easy’ in primary care: learnings from the 2023 Asthma Right Care Summit

**DOI:** 10.1038/s41533-024-00366-x

**Published:** 2024-04-26

**Authors:** Siân Williams, Jaime Correia de Sousa, Ee Ming Khoo, Habib Ghedira, Vincent Mak, Mar Martínez Vázquez, Cláudia Vicente, Darush Attar-Zadeh

**Affiliations:** 1 International Primary Care Respiratory Group, London, UK; 2 Life and Health Sciences Research Institute (ICVS), School of Medicine, University of Minho, Braga, Portugal; 3 Department of Primary Care Medicine, Faculty of Medicine, Universiti Malaya, Kuala Lumpur, Malaysia; 4 Pulmonologist, Lac II, Tunis, Tunisia; 5 International Primary Care Respiratory Group, Tunis, Tunisia; 6 Imperial College Healthcare National Health Service Trust, London, UK; 7 Zorroza Primary Care Unit, Bilbao, Spain; 8 International Primary Care Respiratory Group, Bilbao, Spain; 9 Araceti Family Health Unit, Coimbra, Portugal; 10 International Primary Care Respiratory Group, Coimbra, Portugal; 11 National Health Service North West London Integrated Care Board, London, UK

**Keywords:** Asthma, Health policy, Health services, Patient education, Respiratory signs and symptoms

## Introduction

Asthma affects approximately 262 million people worldwide with an estimated 1000 people dying from asthma attacks each day^1,2^. The majority of asthma attacks are preventable and the substantial mortality, morbidity, healthcare utilisation, environmental and economic burden asthma causes are all largely avoidable. Owing to its impact on the individual and society, asthma warrants a biopsychosocial, holistic approach best provided by primary care. However, the lack of universal health coverage and investment in primary care creates preventable harm, inequalities and inequity within and between countries^3,4^.

Despite knowledge about how to manage it effectively, asthma is often overlooked as a clinical, health and research priority. Currently, a key problem in asthma management is the over-reliance on episodic care defined as a system-wide over-reliance on symptom relief and rescue. This includes inhaled short-acting β_2_-receptor agonists (SABAs), systemic steroids and the overuse of emergency services and hospitalisation, which may partly be caused by lack of adherence to the appropriate medication and disregard of symptoms by patients.

Since 2019, the Global Initiative for Asthma (GINA) no longer recommends treatment of asthma with SABA monotherapy in adults, adolescents and children over 6 years. For the best outcomes, inhaled corticosteroids (ICS)-containing treatment should be initiated when (or as soon as possible after) asthma is diagnosed. All patients should also be provided with a reliever inhaler for quick symptom relief, preferably an anti-inflammatory reliever (AIR), including ICS-formoterol and ICS-SABA^5^.

In 2017, the International Primary Care Respiratory Group (IPCRG) initiated a social movement, Asthma Right Care, to mobilise stakeholders firstly to acknowledge that problems exist, particularly over-reliance on SABA, and secondly to take responsibility for remedying them^3^. This movement aims to disrupt the current system by demonstrating the scale of the problem, and then build on adult-learning principles, offering problem-based education about right care^6,7^, guided by national guidelines and/or GINA.

## Making Asthma Right Care ‘easy’ in primary care

**Fig. 1:**
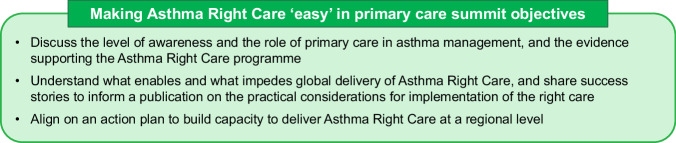
Objectives from the ‘Making Asthma Right Care “easy” in primary care’ summit.

To obtain a greater understanding of current asthma management worldwide, a survey (see Supplementary Information) based on the IPCRG’s situational analyses for its Teach the Teacher^®^ programmes and structured according to IPCRG’s eight person-centred statements (https://www.ipcrg.org/asthmarightcare/what-does-good-quality-asthma-care-look-like, see Supplementary Information) was sent out as an online form to representatives from 47 countries where IPCRG had active contacts with practising clinicians. There was representation from low-, middle- and high-income countries and, in some cases, there was more than one response from a country where different disciplines were represented, including family medicine, community pharmacy and pulmonology (seeing primary care patients). We received 57 responses from 33 countries: 40/57 from respiratory-interested clinicians working in primary care and 17/57 from respiratory specialists. Following the analysis of responses, on the 9 September 2023, an international summit facilitated by a multinational IPCRG faculty took place in Milan, Italy, called ‘Making Asthma Right Care “easy” in primary care’. IPCRG primary care colleagues familiar with Asthma Right Care were joined by 19 delegates from 13 countries from Asia, Latin America and Africa who had expressed interest in engaging in the movement. Figure 1 highlights the objectives of the summit.

## Current asthma management worldwide

The survey responses reported a variation in asthma care, diagnosis and management worldwide—the responses to all questions and representativity from countries are available in the Supplementary Information.

**Fig. 2:**
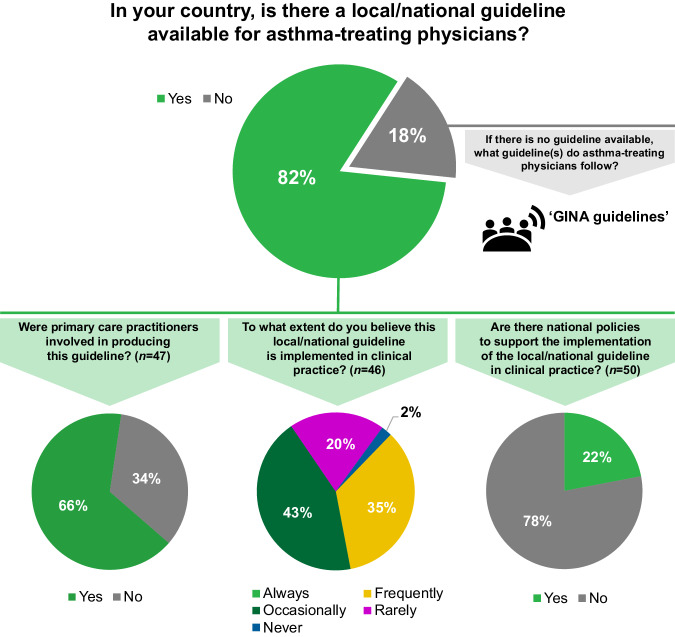
Making Asthma Right Care ‘easy’ in primary care survey results. *N*=57 respondents from 33 countries, unless otherwise stated. The responses include representation from low- (1/33), lower-middle- (7/33), upper-middle- (12/33) and high- (13/33) income countries, according to the World Bank classification^17^. GINA Global Initiative for Asthma.

Overall, 47/57 (82%) respondents reported the existence of local or national guidelines for asthma management (Fig. 2) and about one third of them considered that these were frequently implemented in practice. Approximately three quarters of the respondents felt that there were no national policies to support the implementation of local/national guidelines.

**Fig. 3:**
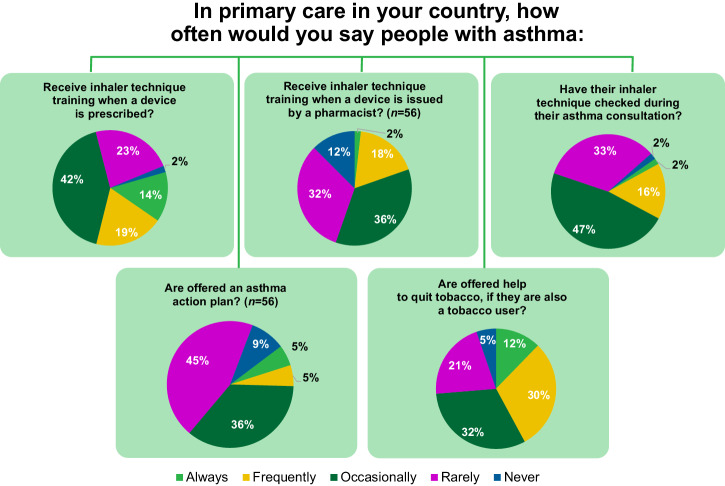
Making Asthma Right Care ‘easy’ in primary care survey results. *N*=57 respondents from 33 countries, unless otherwise stated. The responses include representation from low- (1/33), lower-middle- (7/33), upper-middle- (12/33) and high- (13/33) income countries, according to the World Bank classification^17^.

Key elements of right care were reported missing in many of the countries (Fig. 3). Only approximately a third of respondents noted that asthma inhaler technique training is given always or frequently in their country when a device is prescribed.

## Identifying the key barriers to the delivery of right care

Given time constraints, the meeting mainly focused on asthma management, but diagnostic challenges were also acknowledged as significant. During small-group discussions, delegates considered the drivers and barriers for implementation of right care in their countries. These can be characterised as lack of: (1) education and awareness; (2) capacity and investment in publicly funded healthcare; (3) access to and affordability of medicines; and (4) optimised systems.

There was a strong consensus that improved context-specific asthma education is essential. Some primary care physicians do not consider asthma an important condition that warrants their time to learn about or manage. There is a lack of incentive and of confidence to diagnose asthma in some regions and, in general, confidence and capability in asthma management is too variable to ensure right care for all patients. Family physicians, nurses, pharmacists and patients need to receive better education to recognise when asthma is poorly controlled, to understand the need to treat the underlying inflammation and the risks of over-reliance on SABA monotherapy. Overcoming a lack of disease awareness through education is particularly important in countries where the term ‘asthma’ is not used owing to cultural stigma; alternative incorrect terms such as ‘allergic bronchitis’ contribute to misunderstanding and incorrect treatment. Moreover, appropriate education could facilitate the involvement of people with asthma in their treatment decisions. There is an additional wider need to educate the public and those with the power to influence the public (e.g. journalists, who frequently use wrong inhaler images—for a selection of appropriate images visit: https://www.ipcrg.org/gallery). Educational interventions should be tailored to different levels of asthma awareness and literacy, and the messages should be framed appropriately; for example, emphasis on patient safety is a key element in clinician education, discussions with health administrators and managers, or when speaking with patient organisations. Teachers with the capability to teach primary care and with the knowledge about right care are needed to lead the improvement.

The lack of capacity, resources and time remain universal issues in primary care. Family physicians and nurses often manage many different health conditions, so chronic respiratory diseases are not always a priority. An insufficient number of skilled professionals (i.e. physicians, nurses, pharmacists) able to diagnose asthma is a major barrier in most countries. This can also lead to pulmonologists feeling overburdened.

Ideally, investment in publicly funded healthcare should be a focus to ensure equal access to diagnostic skills, tools and treatments across sectors. Economic constraints and affordability of asthma treatments are major barriers; many countries lack reimbursement schemes and/or apply prescription charges for treatments. The goal should be for universal health coverage to include evidence-based treatment options for asthma.

Continuity of care is key for successful management of asthma. Where primary care is empowered to deliver asthma care, electronic patient records and systems to invite patients at highest risk for follow-up appointments are essential but are not always available. Effective referral systems and compatible electronic patient records are also vital to achieving optimal patient-centred communication between primary, secondary and tertiary care. Depending on the region, access to secondary care may be restricted or permission from respiratory specialists to initiate or change treatment in primary care may be required, which can delay care and compromise safety. Since SABA is available over the counter or can easily be bought online without a prescription in many countries, patients may bypass medical care and self-manage. Optimising systems could facilitate the implementation of guidelines in clinical practice and ensure the delivery of right care.

## Tools to improve asthma care

**Fig. 4:**
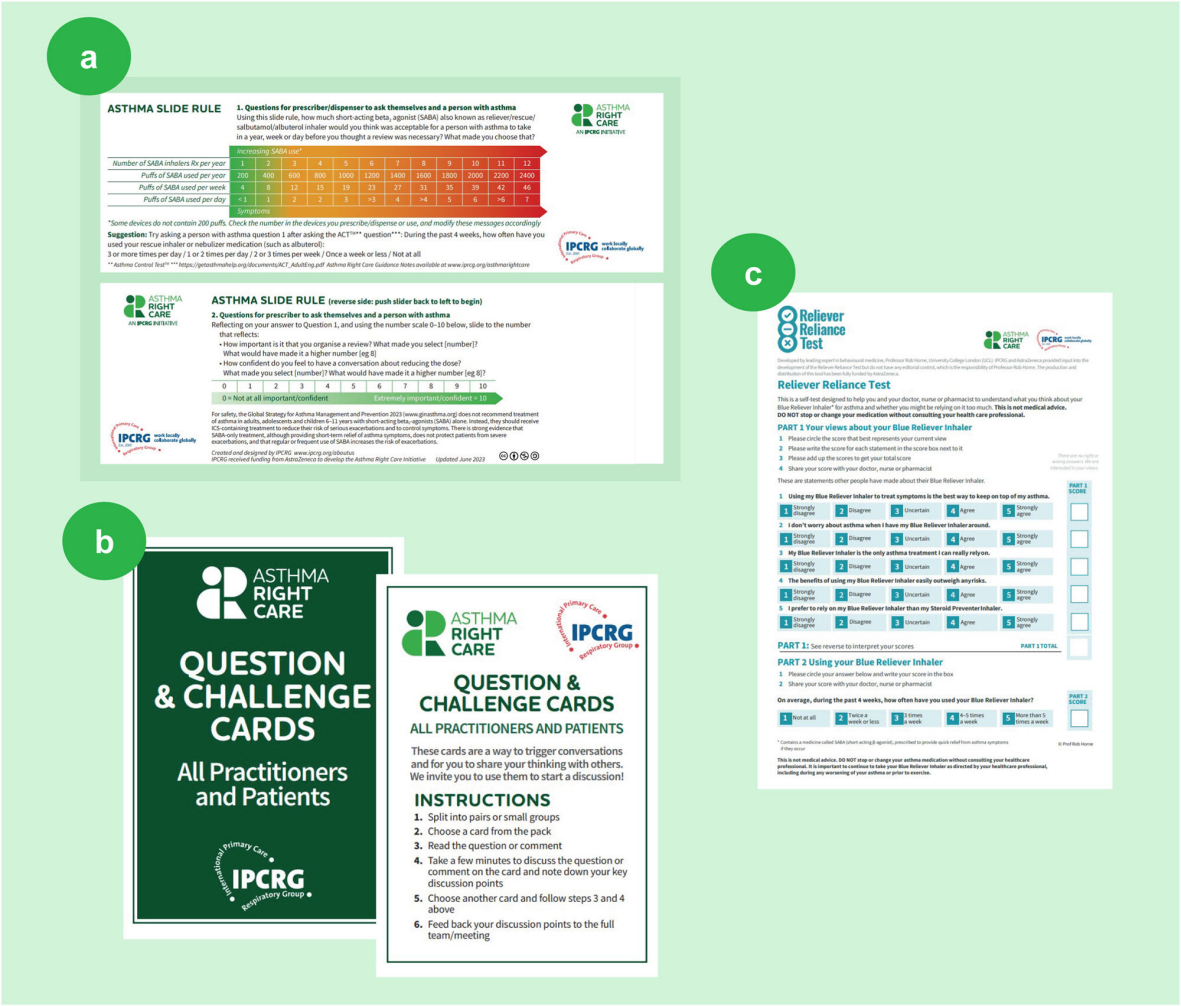
Asthma Right Care tools. **a** Asthma SABA slide rule; **b** ‘Question and challenge’ cards; **c** Reliever Reliance Test. IPCRG. Asthma Right Care Key Resources. Available at: https://www.ipcrg.org/asthmarightcare/asthma-right-care-key-resources (Accessed February 2024). IPCRG International Primary Care Respiratory Group, SABA short-acting β_2_-receptor agonist.

Social movements mobilise followers by prompting conversations that raise awareness of the problem and seek solutions^8^. In small groups divided by region and language and facilitated by IPCRG, the delegates reviewed three Asthma Right Care tools that the IPCRG has developed to facilitate these conversations (Fig. 4). All three tools can be used in clinical practice and also clinical education settings to start conversations that begin to shift perceptions about either a problem or the potential solutions^9^.

(a) **Asthma SABA slide rule**: invites the user to explore how many puffs (as opposed to doses) of SABA inhaler are being used compared with the international guideline advice. Inspired by the Readiness Ruler, on the reverse are a visual analogue scale and Motivational Interviewing questions exploring the importance of requesting a review and confidence to have conversations with healthcare professionals^3^.

(b) **‘Question and challenge’ cards**: useful cards for icebreakers, discussion fora and social media, inspired by the ‘Whose Shoes’ game^3^ (http://nutshellcomms.co.uk/).

(c) **Reliever Reliance Test**: a self-administered test based on the Beliefs About Medicines Questionnaire^10^ and SABA Reliance Questionnaire, co-developed with behavioural scientists, which aims to identify patients at risk of over-reliance on SABA medication and elicit their beliefs^11^.

Since 2017, IPCRG Asthma Right Care country programmes have tested these tools in multiple settings, adapting them in context. More recently, additional tools have also been co-developed and can be shared to help drive change^12^.

## Initiatives to improve asthma care

In addition to reviewing the tools, summit delegates considered the feasibility of adapting several success stories in their countries.

### Three-step AIR Treatment Guideline in New Zealand^13^

**Fig. 5:**
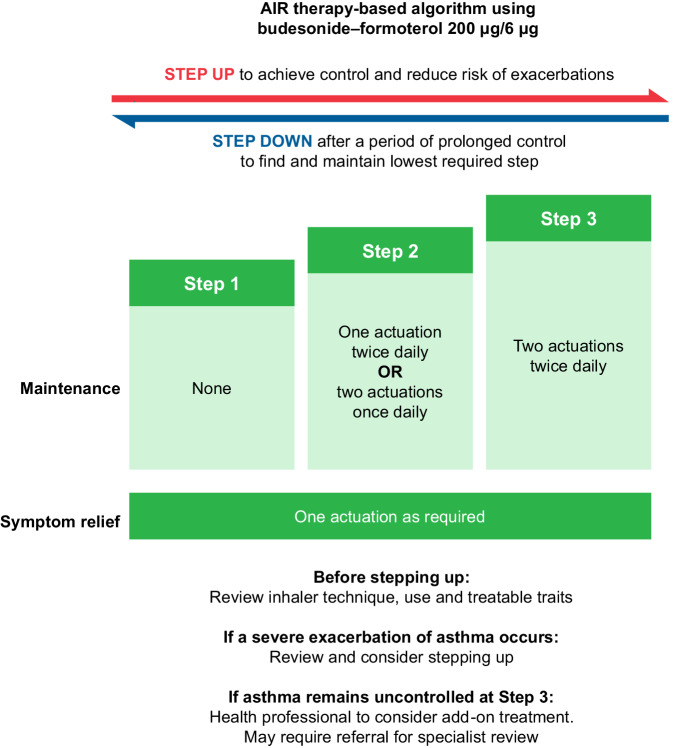
2020 New Zealand AIR therapy-based algorithm. Reproduced with permission from: Asthma and Respiratory Foundation NZ, New Zealand Adolescent and Adult Asthma Guidelines 2020^13^. AIR anti-inflammatory reliever.

In 2020, New Zealand national asthma guidelines, which had strong primary care involvement, recommended AIR therapy as the preferred management approach (Fig. 5). The guidelines were launched with a structured communication plan for wide distribution and encouragement for implementation in practice. A recently published evaluation of the impact of these guidelines identified a significant increase in the dispensing of ICS–formoterol and a reduction in the dispensing of SABA inhalers since the release of the recommendations^14^. This evidence suggests that widespread transition to AIR therapy regimens as recommended by GINA could be achieved if recommended in national asthma guidelines, jointly developed and endorsed as the preferred therapeutic approach by primary and secondary care, and supported by optimised systems for access to medicines and appropriate clinician reimbursement. It is also important to consider the patients’ preference as it will likely impact their adherence to the medication.

### Taking advantage of teachable moments in Spain

In Spain, community pharmacist capability and confidence have been improved through a Teach the Teacher^®^^15^ programme led by IPCRG-taught pharmacists and family physician teachers. This recognises the opportunity for community pharmacists when a SABA inhaler is requested over the counter to take advantage of the teachable moment that it might offer. Strong relationships among patients, family physicians and community pharmacists have been developed to change the asthma pathway, moving away from providing SABA canisters on demand over the counter in pharmacies to using Asthma Right Care tools with individuals, offering advice about right care and prompting those with poorly controlled asthma to visit their family physician for review^16^. This approach resulted in 500,000 fewer SABA canisters sold in 2020 compared with 2018.

### Patient and public engagement in Portugal

In Portugal, creative bottom-up approaches to patient and public engagement have been used. To date, more than 50 organised walks and talks (‘Caminhasma’, meaning ‘walk with asthma’) planned by primary care physicians, nurses and community pharmacists involving almost 4000 people within their communities, have taken place to improve asthma literacy and awareness. Subsequently, the initiative has been replicated in Brazil. Also, the Asthma Right Care (known as ‘CAPA’) team has co-created a teaching film and delivered a series of television interviews for a health channel, as well as clinical webinars. A new campaign aimed at adolescents with a video promoting a game between the viewers to teach about asthma is being developed in partnership with the Ministry of Education of Portugal.

### Draft national asthma law in Argentina

In Argentina, respiratory-interested clinicians have advocated to the national senate for an asthma law to allow equal access to care for every person with asthma. They have separately worked with colleagues in other specialities to raise awareness about specific at-risk groups such as pregnant women who, according to an unpublished national survey, often stop taking their asthma prescription during pregnancy.

### Asthma lexicon in Tunisia

In Tunisia, a multidisciplinary Asthma Right Care steering group prioritised a SABA overuse awareness programme in community pharmacies supported by the Pharmacists Union and the Private Physicians Union. The programme was presented to the Minister of Health who encouraged the initiative. IPCRG’s tools, such as the Reliever Reliance Test and Asthma Slide Rule, were translated to Tunisian dialect to support the nationwide programme. The group also developed a lexicon of Tunisian dialect’s usual asthma and allergy words with their translation in French and English. This lexicon ensures that the terminology used in communication becomes more consistent across the healthcare system.

Many other success stories are emerging, demonstrating the value of bottom-up approaches that engage primary care and patients in highlighting the problem of episodic care and then taking responsibility to address it through education and advocacy for system change.

### Nine statements to improve respiratory care

**Fig. 6:**
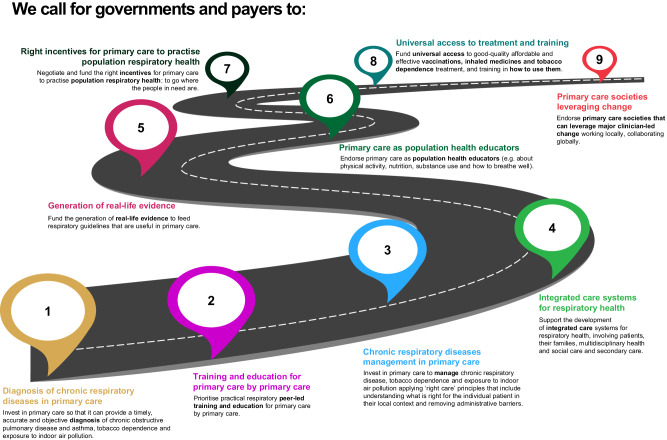
Nine statements agreed with the IPCRG and WONCA Europe to improve respiratory care. IPCRG International Primary Care Respiratory Group, WONCA World Organization of Family Doctors.

The delegates were shown and supported nine key actions to improve respiratory care that IPCRG and the World Organization of Family Doctors (WONCA) Europe agreed at the 2023 WONCA Council meeting (see Fig. 6).

## Conclusions

Currently, no universal approach exists to tackle the obstacles to right care in asthma, but the Asthma Right Care movement has demonstrated that change is possible with leadership, teamwork, community involvement and commitment. There are a number of tools available that can be used and adapted considering local contexts. To achieve large-scale improvements, context-specific strategies that engage as many parts of the healthcare system in as many geographic areas as possible, are needed; the aim is for improved awareness and behaviour change. At the end of the summit, it was agreed that to generate and sustain change every country needs ‘Asthma Right Care champions’ passionate about engaging all stakeholders using Asthma Right Care tools. The IPCRG commits to building these champions’ capacity to advocate for and lead change, and to teach their peers through IPCRG Teach the Teacher^©^ cascade models^15^.

### Reporting summary

Further information on research design is available in the Nature Research Reporting Summary linked to this article.

### Supplementary information

Supplementary Information

Reporting Summary

Dataset

## Data Availability

All data supporting the findings presented in this manuscript are available within the paper and its Supplementary Information.
