# Supplementary material for: How to make Asthma Right Care ‘easy’ in primary care: learnings from the 2023 Asthma Right Care Summit
Source: NPJ Prim Care Respir Med. 2024 Apr 26;34:4. doi: 10.1038/s41533-024-00366-x (PMC11053093; doi:10.1038/s41533-024-00366-x)
Supplement: Supplementary file 1 — Supplementary Information [file 41533_2024_366_MOESM1_ESM.pdf]

Supplementary Notes

The survey below was sent out to IPCRG member countries and delegates attending the 'Making Asthma Right Care "easy" in primary care' summit, to obtain a greater understanding of current asthma management worldwide.

Representing your country in general, rather than your personal behaviour/experience, please answer the following questions focussing on the publicly funded health sector, or the health sector serving the majority in your country.

1) Please enter your country and affiliation. [Free text]
2) In your country, is there a national strategy for non-communicable diseases? [Yes/No]
   a. Is asthma included in the national strategy for non-communicable diseases? [Yes/No]
3) Regarding asthma diagnosis in general, how usual is it for asthma to be diagnosed in primary care? [Always / Frequently / Occasionally / Rarely / Never (it is a secondary care role)]
4) Regarding asthma diagnosis in general, how does diagnosis in primary care include an objective measure (e.g. peak flow, spirometry, other)? [Always / Frequently / Occasionally / Rarely / Never]
      i. If used, please specify which measure. [Free text]
5) Regarding asthma diagnosis in general, how often is asthma diagnosed over several consultations? [Always / Frequently / Occasionally / Rarely / Never]
6) In your country, typically what medicines are the majority of primary care practitioners permitted to prescribe for the management of mild to moderate asthma (multiple responses allowed)? [ICS + LABA / ICS + LABA + SABA / ICS + SABA / LTRA / MART / OCS / SABA monotherapy / SMART / Other]
7) Thinking about the majority of primary care doctors in your country, are there any barriers to people with asthma accessing these treatments (e.g. financial burden, restrictions on the number of prescriptions of ICS or MART inhalers per year per patient/clinic, permission required from respiratory specialists to initiate treatment, availability of medicines)? [Yes/No]
8) What are the barriers to people with asthma accessing these treatments?
9) Typically, how often do people with asthma participate in the choice of inhaler devices for managing their asthma? [Always / Frequently / Occasionally / Rarely / Never]
   a. Are there any barriers to involving people with asthma in treatment decisions? [Yes/No]
      i. What are the barriers to involving people with asthma in treatment decisions? [Free text]
10) In primary care in your country, how often would you say people with asthma:
   a. Receive inhaler technique training when a device is prescribed? [Always / Frequently / Occasionally / Rarely / Never]
   b. Receive inhaler technique training when a device is issued by a pharmacist? [Always / Frequently / Occasionally / Rarely / Never]
   c. Have their inhaler technique checked during their asthma consultation? [Always / Frequently / Occasionally / Rarely / Never]
   d. Are offered an asthma action plan? [Always / Frequently / Occasionally / Rarely / Never]
   e. Are offered help to quit tobacco, if they are also a tobacco user? [Always / Frequently / Occasionally / Rarely / Never]

11) In general, in your country, how often are people with asthma followed up consistently by the same team? [Always / Frequently / Occasionally / Rarely / Never]
   a. What is the typical frequency of follow-up? [Every 3–4 months / Every 6 months / Every 12 months / On patient demand / Only in emergency / Other (please specify)]
   b. What are the barriers and solutions to continuity of care (e.g. shared electronic patient record, patient-held record, call and recall systems)? [Free text]
   c. In primary care, is there a preferred structure for asthma reviews? [Yes/No]
      i. What is the preferred structure for asthma reviews? [Free text]
12) In general, are people with asthma referred in a timely manner to secondary healthcare professionals with a respiratory specialism when their symptom control is poor and cannot be managed in primary care? [Always / Frequently / Occasionally / Rarely / Never]
   a. What are the normal processes in place to enable referral in a timely manner to secondary healthcare professionals when their symptom control is poor and cannot be managed in primary care? [Free text]
   b. If not, what are the existing barriers to referring people with asthma in a timely manner to secondary healthcare professionals when their symptom control is poor and cannot be managed in primary care? [Free text]
13) In your country, is there a local/national guideline available for asthma-treating physicians? [Yes/No]
   a. If there is no local/national guideline available in your country, what guideline(s) do asthma-treating physicians follow?
   b. If there is, please provide the URL and date of the current local/national guideline [Free text]
      i. Were primary care practitioners involved in producing this guideline? [Yes/No]
   c. To what extent do you believe this local/national guideline is implemented in clinical practice? [Always / Frequently / Occasionally / Rarely / Never]
      i. What are the challenges to implementing this guideline in clinical practice, if any? [Free text]
   d. Are there national policies to support the implementation of the local/national guideline in clinical practice (e.g. targets/incentives, electronic data, restrictions on the number of prescriptions of certain medications per year, making certain medications, such as oral steroids and inhaled SABA, unavailable over the counter)? [Yes/No]
      i. What are these policies? [Free text]
      ii. What policies could be useful? [Free text]
14) To what extent do you believe that the GINA 2023 track 1 recommendations are implemented in clinical practice in your country? [Always / Frequently / Occasionally / Rarely / Never]
   a. Are there any data to support your view? [Free text]
   b. What are the challenges to implementing GINA 2023 track 1 recommendations in clinical practice in your country, if any? [Free text]
15) To what extent do you believe that the GINA 2023 track 2 recommendations are implemented in clinical practice in your country? [Always / Frequently / Occasionally / Rarely / Never]
   a. Are there any data to support your view? [Free text]
   b. What are the challenges to implementing GINA 2023 track 2 recommendations in clinical practice in your country, if any? [Free text]

Survey responses
The complete set of responses and raw data are available as separate files within the Supplementary Information.

Survey participants

Name	Country	Type
Ana Stok	Argentina	IPCRG contact
Carlos Martin Elias Rein	Argentina	Delegate
Sergio Zunino	Argentina	Delegate
Debbie Rigby	Australia	IPCRG contact
Ajay Kumarroy	Bangladesh	IPCRG contact
Monsur Habib	Bangladesh	IPCRG contact
Nazim Uzzaman	Bangladesh	IPCRG contact
Sónia Martins	Brazil	IPCRG contact
Alan Kaplan	Canada	Delegate
Chunhua Chi	China	IPCRG contact
Jaime Gómez Ayala	Colombia	Delegate
Luis Ugalde	Costa Rica	Delegate
Josephine Vargas	Dominican Republic	IPCRG contact
Haytham Diab	Egypt	Delegate
Ayman Farghaly	Egypt	Delegate
Izolde Bouloukaki	Greece	IPCRG contact
Ioanna Tsiligianni	Greece	Delegate
Monica Barne	India	IPCRG contact
Andrew Owuor	Kenya	Delegate
Ee Ming Khoo	Malaysia	Faculty
Hani Salim	Malaysia	IPCRG contact
Désirée Larenas-Linnemann	Mexico	Delegate
Nora Martínez	Mexico	IPCRG contact
Gloria Pila	Mexico	Delegate
Katarina Stavric	North Macedonia	IPCRG contact
Osman Yusuf	Pakistan	IPCRG contact
Jaime Correia de Sousa	Portugal	Faculty
Cláudia Vicente	Portugal	Faculty
Carmen Busneag	Romania	IPCRG contact
Sergiu Chirila	Romania	IPCRG contact
Adela Iancu	Romania	IPCRG contact
Catalina Panaitescu	Romania	IPCRG contact
Ong Kian Chung	Singapore	IPCRG contact
Žan Trontelj	Slovenia	IPCRG contact
Ismail Kalla	South Africa	Delegate

AyandaTrevor Mnguni	South Africa	Delegate
Marina Garcia Pardo	Spain	Delegate
Mar Martínez	Spain	Faculty
Francesc Moranta Ribas	Spain	IPCRG contact
Miguel Roman Rodriguez	Spain	Delegate
Bjorn Stallberg	Sweden	IPCRG contact
Samuel Chen Hung-Ling	Taiwan	Delegate
Kulachade Gesakomol	Thailand	Delegate
Apichai Wattanapisit	Thailand	IPCRG contact
Asma Ben Brahem	Tunisia	IPCRG contact
Habib Ghedira	Tunisia	Faculty
Umut Gok Balci	Turkey	IPCRG contact
Winceslaus Katagira	Uganda	IPCRG contact
Darush Attar-Zadeh	United Kingdom	Faculty
Katherine Hickman	United Kingdom	IPCRG contact
Garry MacDonald	United Kingdom	IPCRG contact
Vince Mak	United Kingdom	Faculty
Siân Williams	United Kingdom	Faculty
Samir Naik	United Arab Emirates	Delegate
Mazen Zouayhed	United Arab Emirates	Delegate
Stephen Brunton	United States of America	IPCRG contact
Quan Vu Tran Thien	Vietnam	IPCRG contact

IPCRG, International Primary Care Respiratory Group

Asthma Right Care eight person-centred statements

For further information and resources, please visit: https://www.ipcrg.org/resources/search-resources/what-does-good-quality-asthma-care-look-like-pdf

What does good quality asthma care look like?

ASTHMA RIGHT CARE
AN IPCRG INITIATIVE

IPCRG International Primary Care Respiratory Group Est. 2001 — work locally collaborate globally

IPCRG is regularly asked by primary care clinicians to define good quality care. We take the view that primary care is person-centred, and therefore the best way to define quality is from the perspective of the person at risk of, or with the condition. From our regular conversations with expert patients and clinicians **we have summarised what good quality care should look like from a patient perspective and how can clinicians provide that in 8 person-centred statements.** These are divided into four areas: Diagnosis, Management, Review, When control is poor. Our vision is that clinical teams will use them to benchmark their practice and potentially identify an area for improvement. Our own programme of work is steered by these statements. We are currently defining the competencies required to deliver them and the teaching methods and tools to enable delivery.

IPCRG tools that we already offer are listed in green italics.*

People with asthma deserve...

Diagnosis

1 A timely, accurate and formal/objective diagnosis of their asthma by their primary healthcare team. *The 'jigsaw puzzle' approach to building a diagnostic picture of asthma in primary care over time*.

Management

2 To receive adequate inhaler treatment for their asthma according to the best practice recommendations for their level of disease severity. *Asthma Right Care Key Resources*

3 To participate in the choice of treatment for their asthma, including the decision between different options of inhaler devices eg *rightbreathe*

4 To have appropriate inhaler technique training and to agree an asthma action plan shared with their health care providers eg *Inhaler videos*, *Canadian action plan*, *SMART action plan*

5 Counselling and treatment if they are tobacco dependent, a yearly flu vaccination and COVID-19 vaccination *Desktop helper helping people quit*

Review

6 Follow-up appointments at acceptable intervals or after a change in management, for the management of their asthma that must include structured assessment of control eg *ACT, wellbeing & evaluation of future risk*

7 That their difficult-to-manage asthma is evaluated by their primary health care team following a structured approach in order to identify any solvable questions before they are referred to secondary care. *Difficult to manage asthma desktop helper [under review]*

When control is poor

8 To have easy and timely access/referral to a primary or secondary health care professional who is skilful in asthma management whenever their symptoms cannot be self-managed or when their asthma cannot be managed in primary care eg *referral letter*

***Interactive version available with hyperlinks. Scan the QR code.**

Oct 2023

International Primary Care Respiratory Group
IPCRG Est. 2001
work locally
collaborate globally

ASTHMA RIGHT CARE

Supplementary Notes
Survey responses

This document includes the responses from the delegates attending the Asthma Right Care summit in Milan 2023 and from the IPCRG member countries

In your country, is there a national strategy for non-communicable diseases?

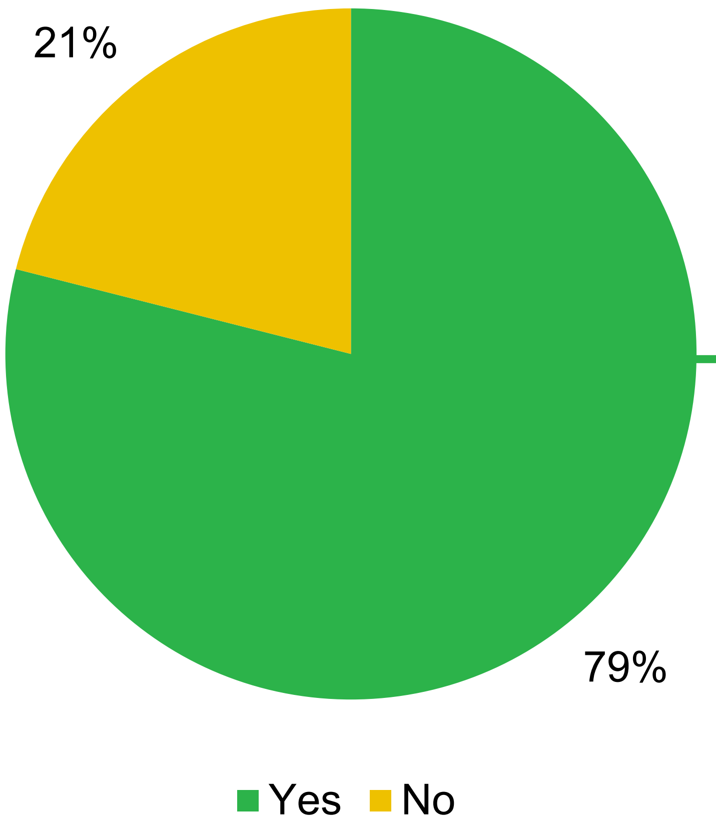

Is asthma included?

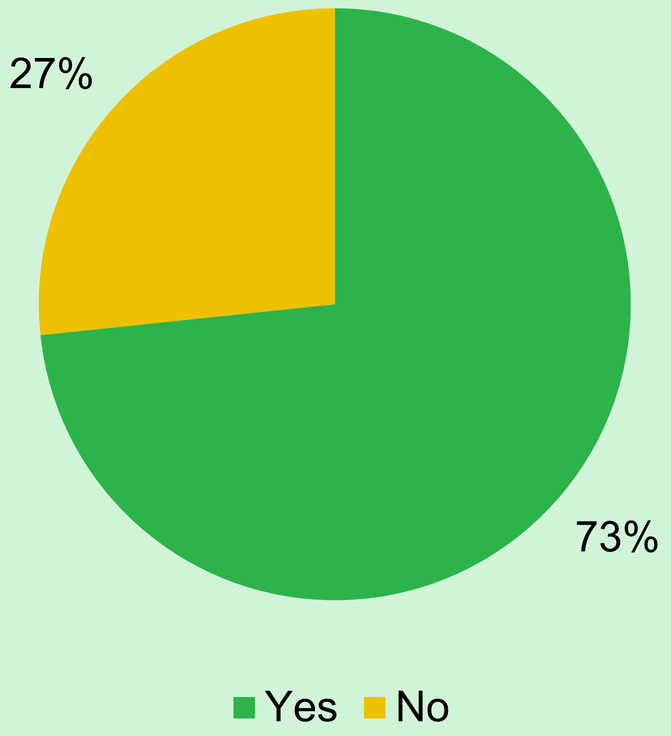

N=57 respondents from 33 countries

Regarding asthma diagnosis in general, how often…

…is asthma diagnosed in primary care?

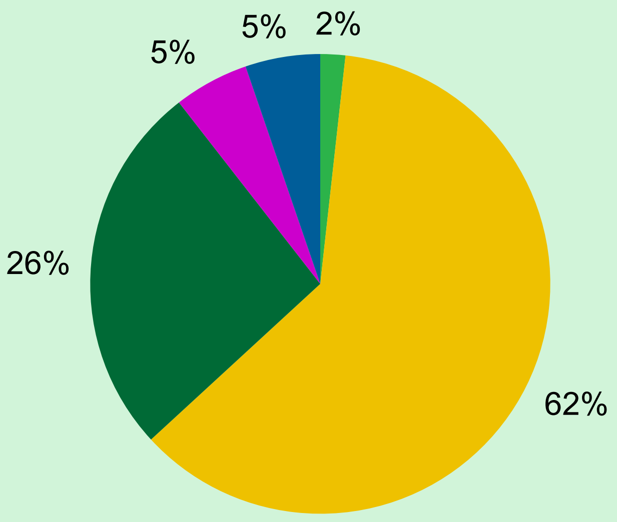

…does diagnosis include an objective measure (e.g. peak flow, spirometry, other)?

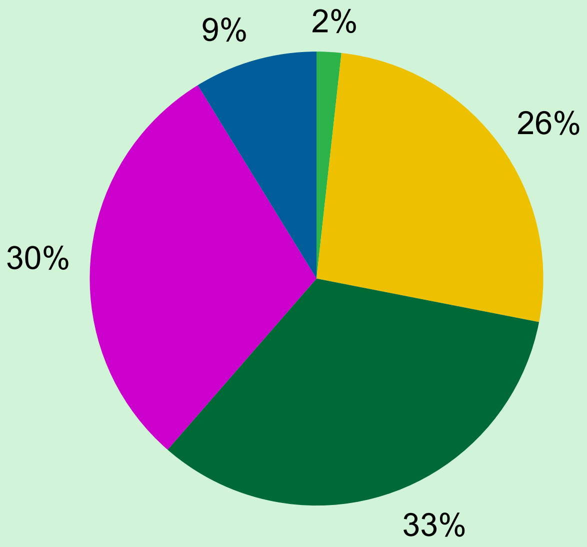

…is asthma diagnosed over several consultations?

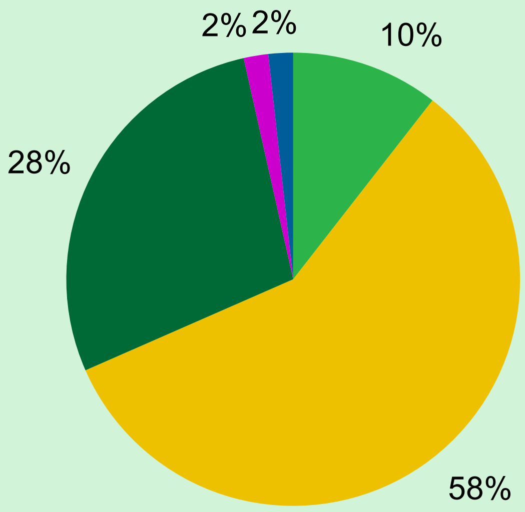

Always | Frequently | Ocasionally | Rarely | Never

N=57 respondents from 33 countries

If an objective measure is used, which one?

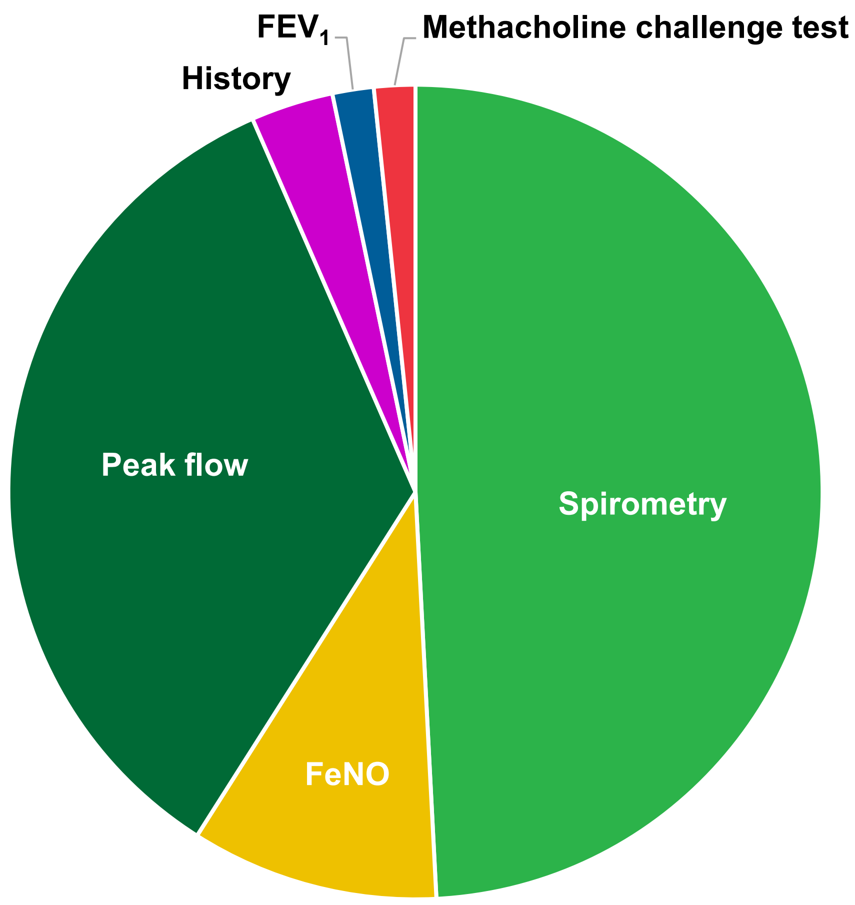

N=57 respondents from 33 countries
FeNO, fractional exhaled nitric oxide; FEV$_1$, forced expiratory volume in the first second

How often is asthma diagnosed in primary care?

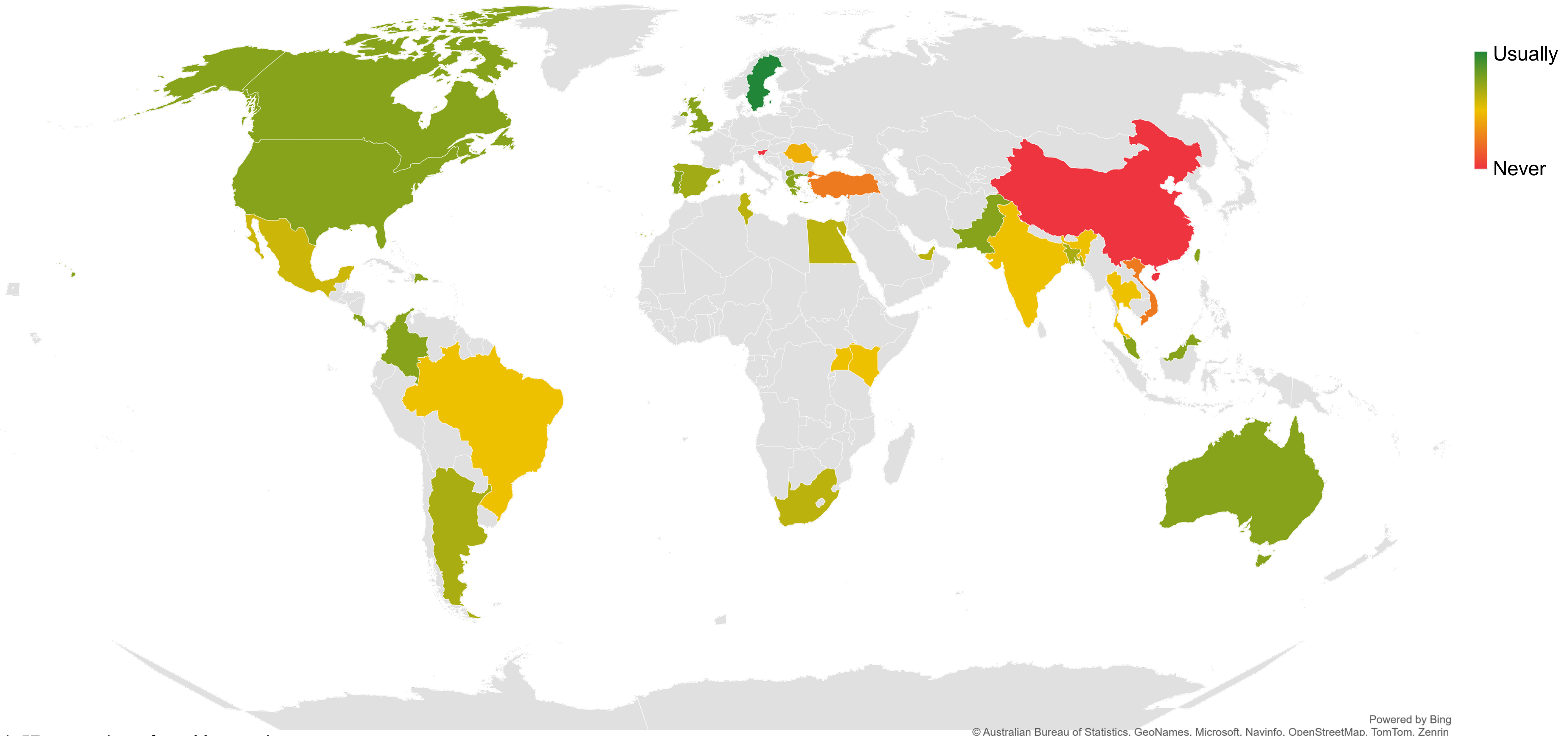

N=57 respondents from 33 countries

In your country, what medicines can primary care practitioners prescribe for the management of mild-to-moderate asthma?

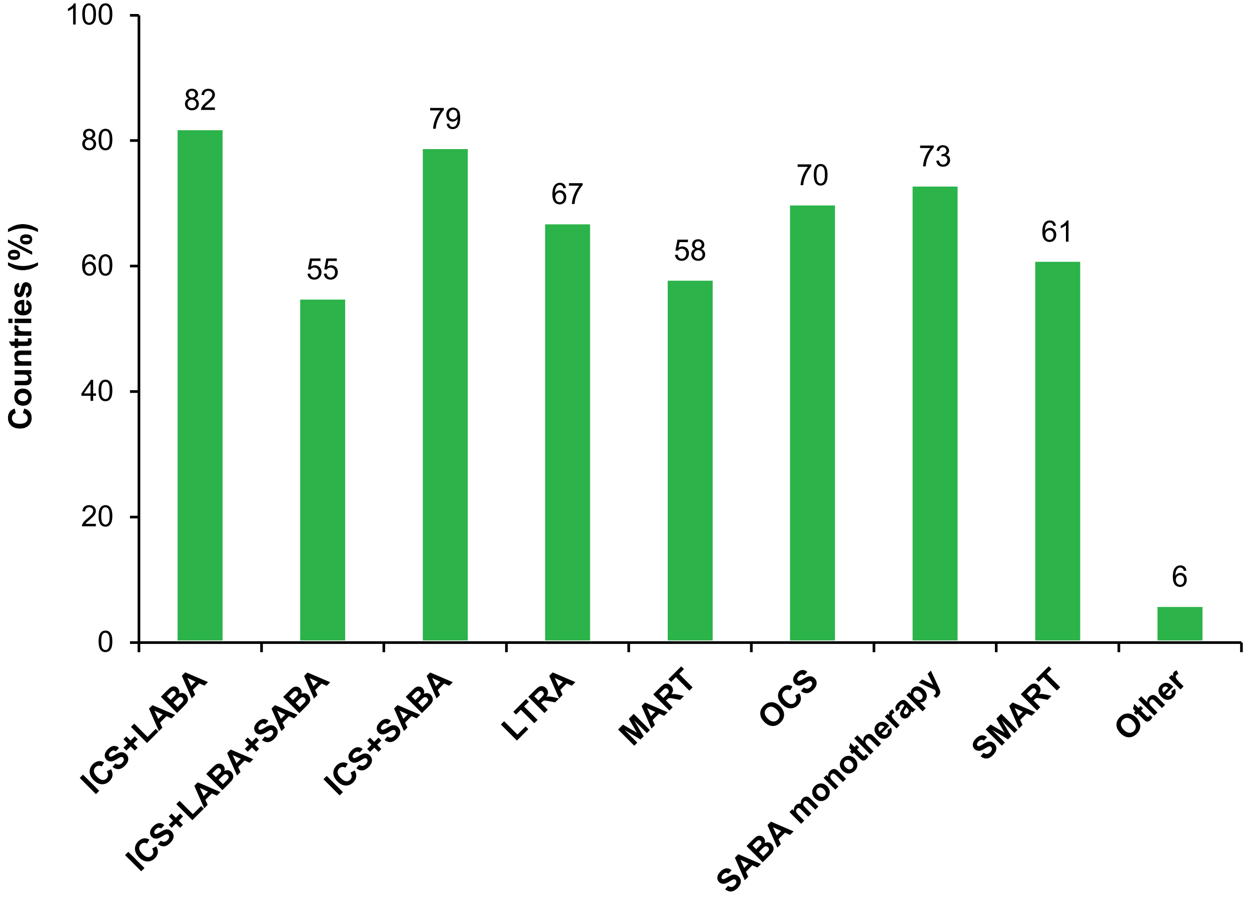

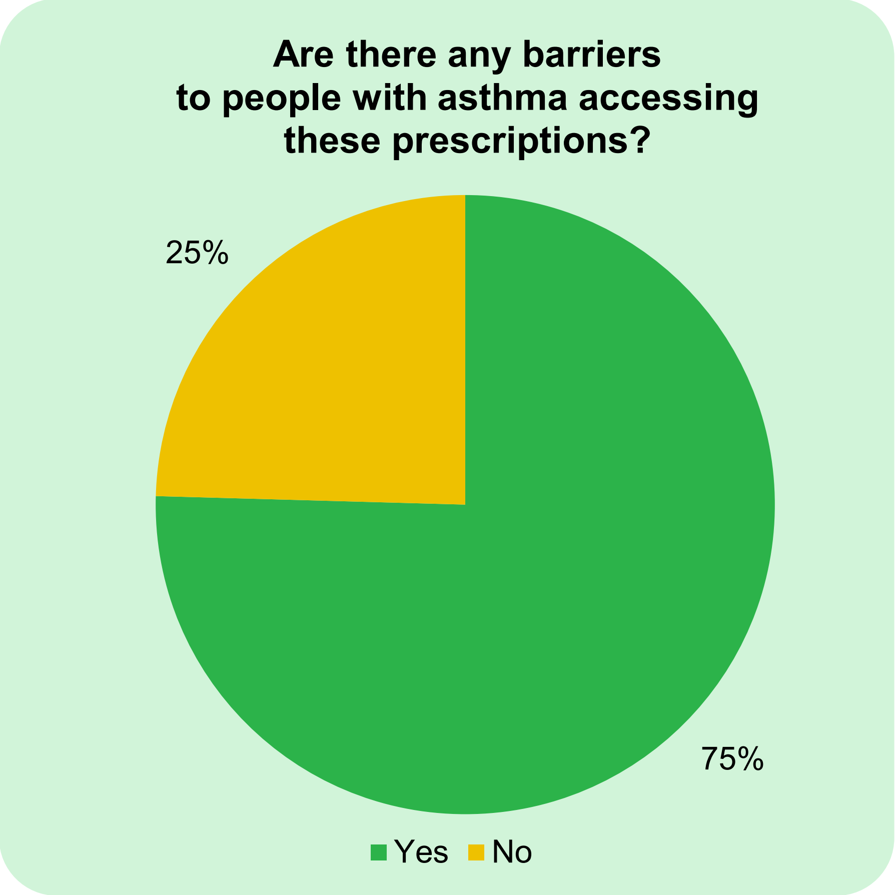

N=57 respondents from 33 countries
ICS, inhaled corticosteroids; LABA, long-acting β_2-receptor agonist; LTRA, leukotriene receptor antagonist; (S)MART, (Symbicort) maintenance and reliever therapy; OCS, oral corticosteroids; SABA, short-acting β_2-receptor agonist

If there are barriers to people with asthma accessing medicines, what are they?

AFFORDABILITY

- **Prescription charges** (UK)
- **Economic constraints** (Argentina, Colombia, South Africa, Canada, Malaysia, Mexico, UAE, Kenya, Costa Rica, Romania, Bangladesh, Uganda, Portugal, USA, Singapore, Vietnam, Pakistan, Dominican Republic, Bangladesh, China, Tunisia)
- **Scheme of treatment plan** (Thailand)
- **Lack of reimbursement** (Canada, UAE, Tunisia, Vietnam)

SOCIAL

- **Social barriers** (Pakistan)

EDUCATION

- **Lack of education** (Canada, Uganda, Bangladesh, India, Romania South Africa)
- **Difficulty to diagnose** (Singapore, North Macedonia)
- **Lack of confidence in primary care practitioners to diagnose asthma** (India)

STRUCTURAL

- **Access to secondary care** (Romania, Malaysia, North Macedonia, Greece)
- **Restrictions on the number of prescriptions of SMART/MART inhalers per year per patient/clinic** (Malaysia)
- **Permission required from respiratory specialist to initiate treatment** (Malaysia, Romania)
- **Lack of availability of medicines** (Malaysia, Argentina, Uganda, China)
- **Only ICS available is beclomethasone 50 µg/puff** (Mexico)

ICS, inhaled corticosteroids; (S)MART, (Symbicort) maintenance and reliever therapy

Typically, how often do people with asthma participate in the choice of inhaler devices for managing their asthma?

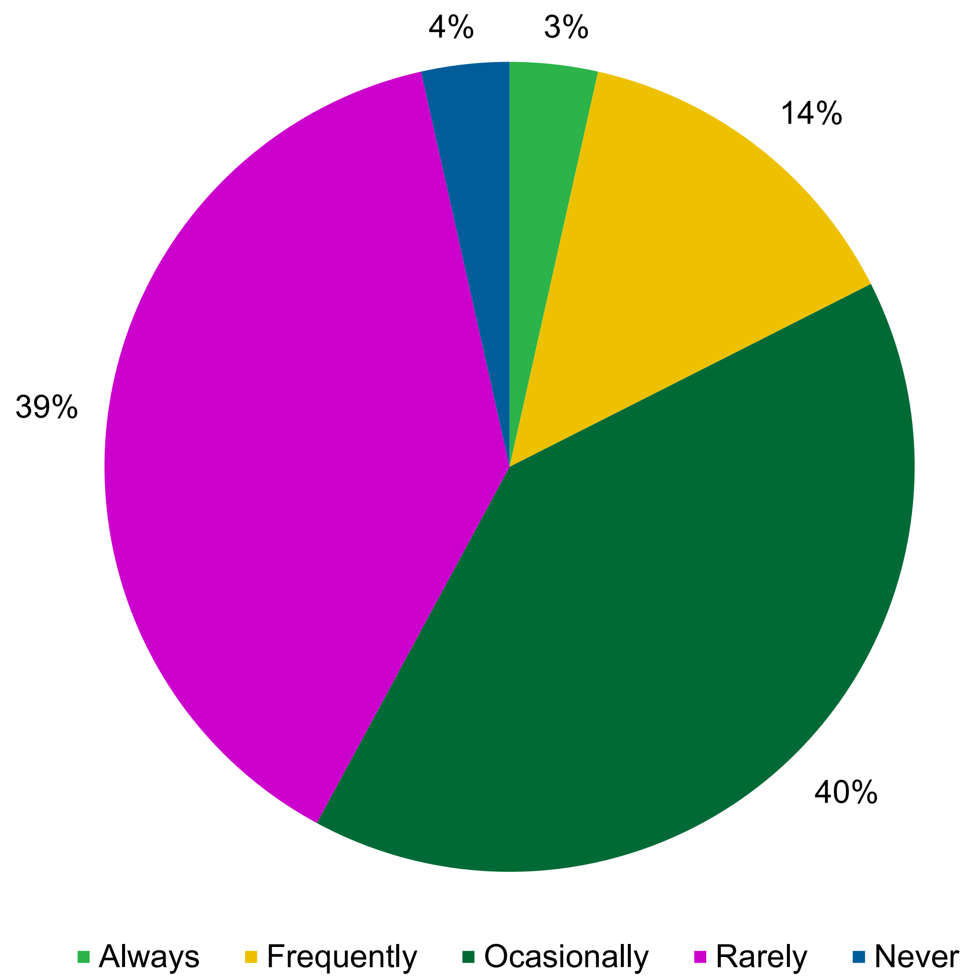

Are there any barriers to involving people with asthma in treatment decisions?

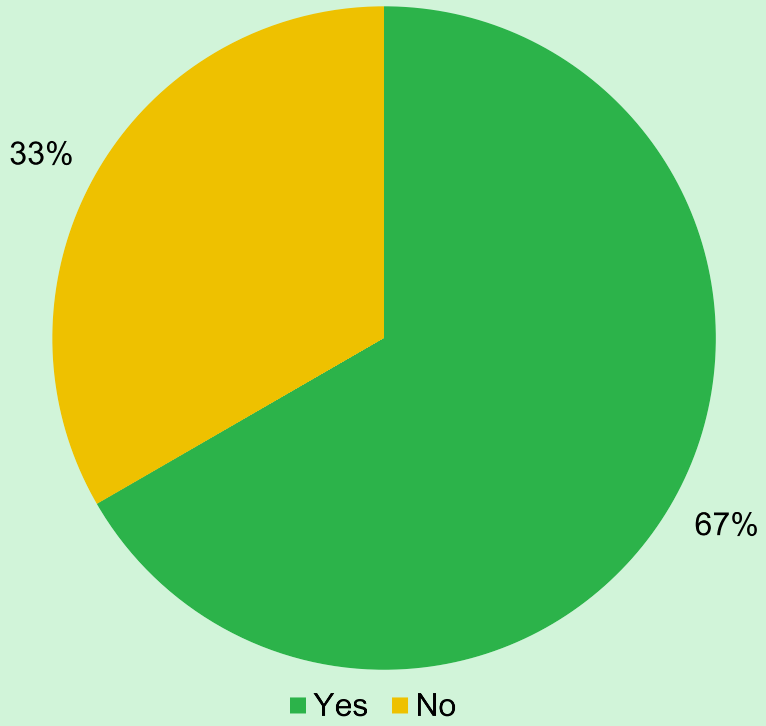

N=57 respondents from 33 countries

If there any barriers to involving people with asthma in treatment decisions, what are they?

CLINICIANS' CAPABILITY

- **Lack of communication skills** (UK, Canada)
- **Lack of team support** (Mexico, Bangladesh, India)
- **Lack of time to involve the patients** (Australia, Romania)

HEALTH LITERACY

- **Poor health literacy** (Malaysia, UAE, Kenya, Uganda, Portugal, Singapore, Greece, Bangladesh, Pakistan)
- **Lack of clinicians' awareness of the different treatment options** (UK, Argentina, Uganda, India)

CULTURE

- **Cultural barriers** (Greece, Taiwan, Spain, Tunisia, Canada, Costa Rica, Pakistan, Brazil, Mexico)

LACK OF OPPORTUNITY

- **Lack of time** (UK, Mexico, Kenya, Bangladesh, Vietnam, Pakistan, India)
- **Lack of availability of medicines to choose from** (Malaysia, Mexico, North Macedonia, Brazil, South Africa)
- **Medication available on the essential drug list** (South Africa)

ASTHMA RIGHT CARE

In primary care in your country, how often would you say people with asthma…

…receive inhaler technique training when a device is prescribed?

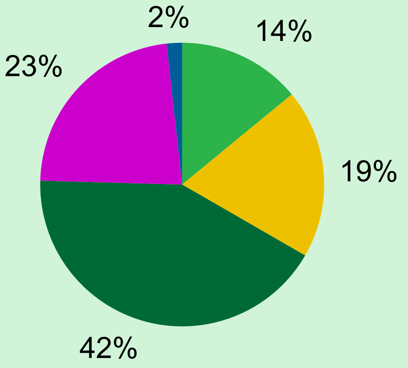

…receive inhaler technique training when a device is issued by a pharmacist? (n=56)

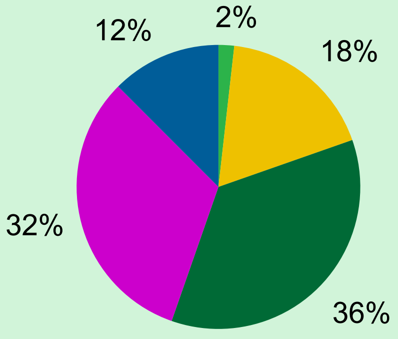

…have their inhaler technique checked during their asthma consultation?

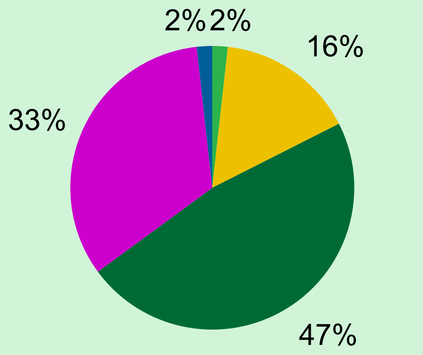

- Always
- Frequently
- Ocasionally
- Rarely
- Never

…are offered an asthma action plan? (n=56)

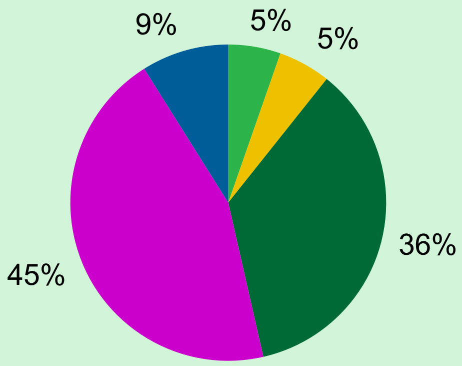

…are offered help to quit tobacco, if they are also a tobacco user?

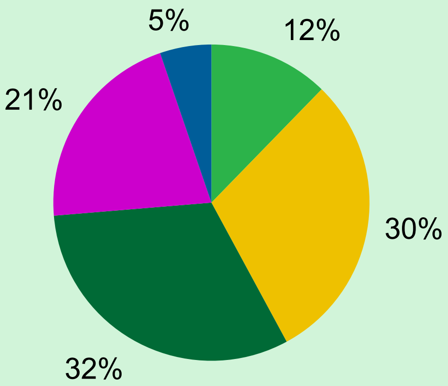

N=57 respondents from 33 countries, unless otherwise stated

In your experience, how often are people with asthma followed up consistently by the same team?

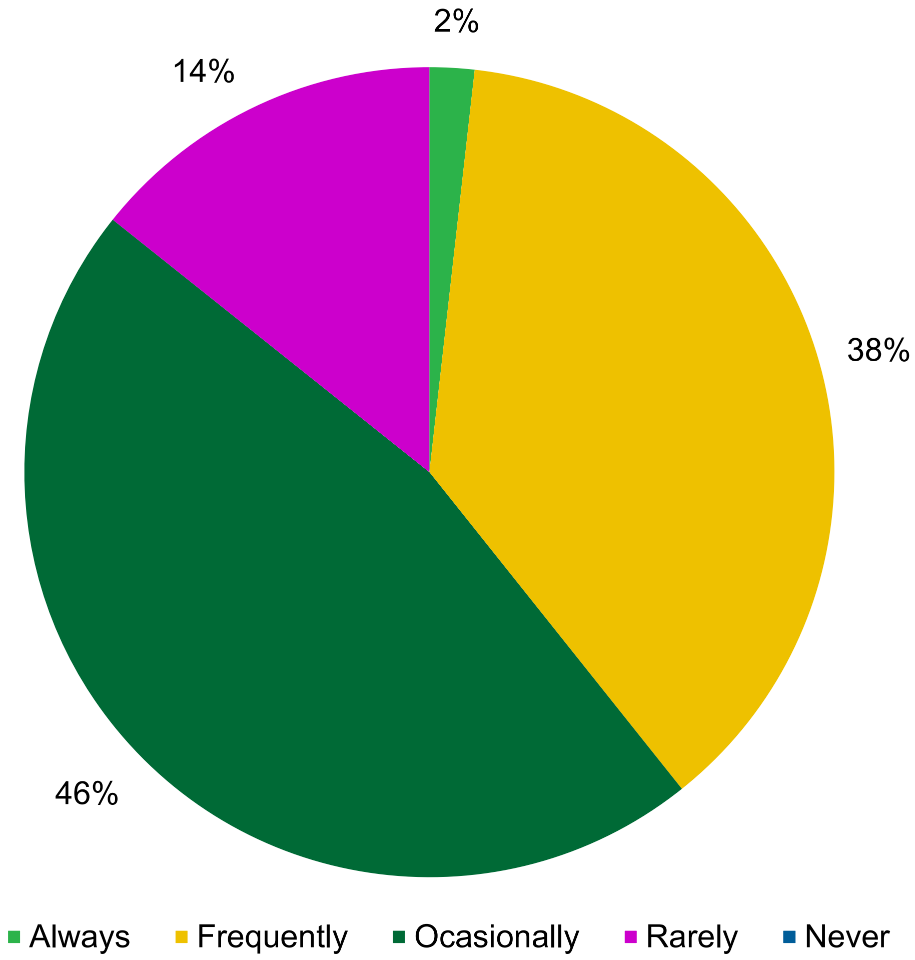

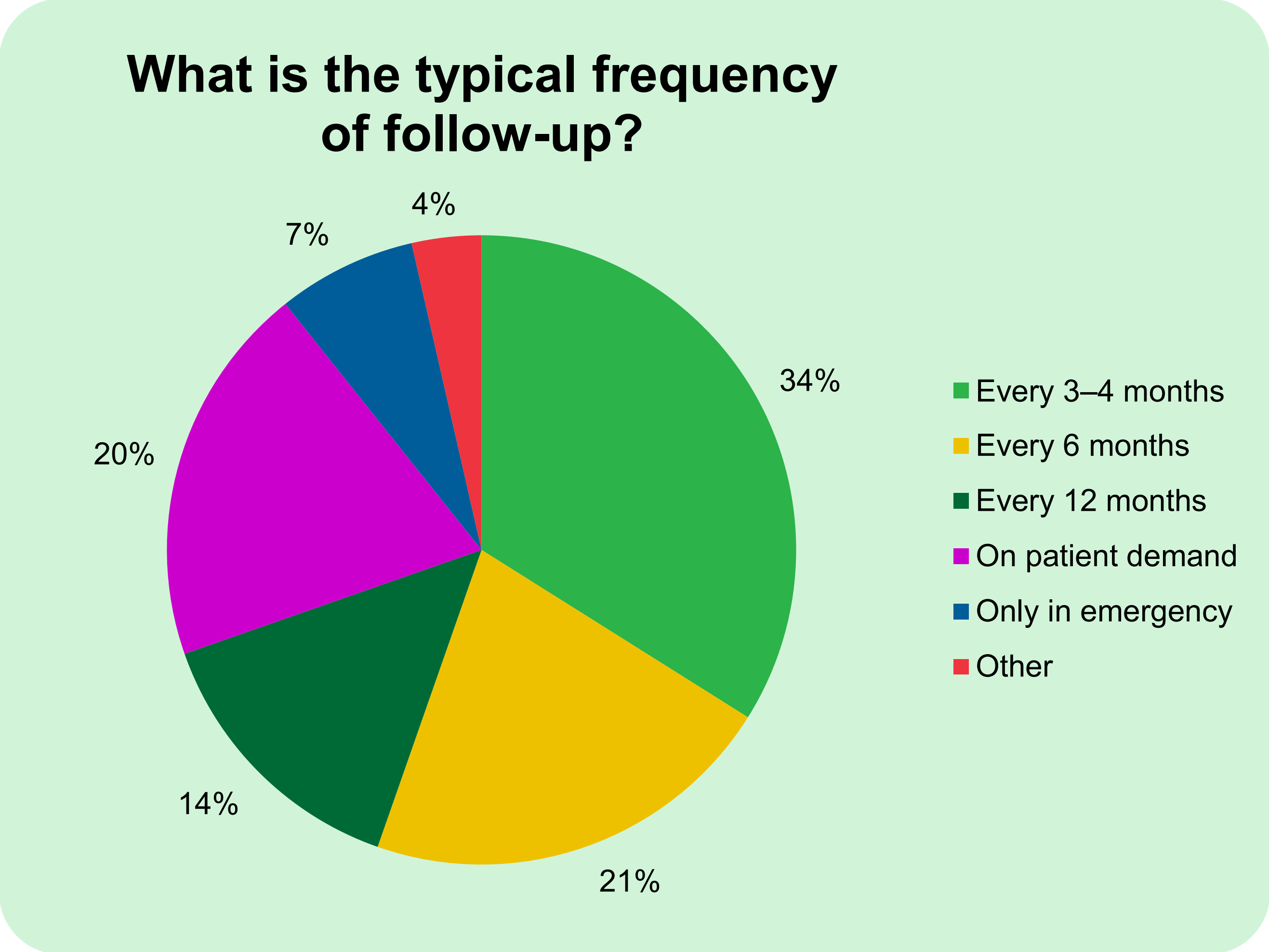

N=56 respondents from 32 countries

What are the barriers and solutions to continuity of care?

BARRIERS

Clinician and patient capability and confidence

- Skill set (UK)
- Communication between primary (including community pharmacist), secondary and tertiary care – electronic records not always compatible (UK, Thailand, Malaysia, Mexico, Kenya, UAE, Argentina, Romania, Singapore)
- Lack of disease awareness / training for continuity of care (Spain, Argentina, North Macedonia)
- Lack of capacity/resources/time (Colombia, North Macedonia, Greece, Canada, Romania, South Africa)
- Lack of patient education (UAE)

Opportunity

- Feasibility (Egypt)
- Financial considerations (Argentina, UK, Uganda, Tunisia)
- No call-and-recall system (Mexico, Romania)
- No patient records (Bangladesh, Vietnam, Pakistan, Mexico, India)
- Patients being followed in hospital vs primary care (Turkey)

Motivation

- Few performance indicators for CRD, inertia and routine (Portugal)
- Reimbursement (Costa Rica)
- Asthma is not considered an important condition by PCPs (Spain)

SOLUTIONS

Clinician and patient capability and confidence

- Patient and clinicians' education (Portugal)
- Capacity-building of GPs across the country (Bangladesh)
- Work with nurses and pharmacists (Portugal)
- Outreach to patient groups, community engagement (Bangladesh)
- Alams on the patient record (Romania)

Opportunity

- Call-and-recall systems (Taiwan, Romania, Spain)
- Shared electronic records (Egypt, Portugal, Tunisia)
- More integrated primary care system (South Africa)
- Asthma clinics, asthma protocol / care pathway, asthma registry (Malaysia)
- Asthma action plans (Portugal)
- Need for primary care teams (Malaysia, USA)
- Call system to remind the patient of the follow-up appointment; have the patient reachable for home visits (Dominican Republic)
- Funding for asthma review by GPs (Australia)
- Promotion of pharmacist-led asthma medication reviews (Australia)

Motivation

- Monthly ACT results with prompts for GPs if the results drop (Slovenia)

ACT, Asthma Control Test; CRD, chronic respiratory disease; GP, general practitioner; PCP, primary care practitioner

Is there a preferred structure for asthma reviews?

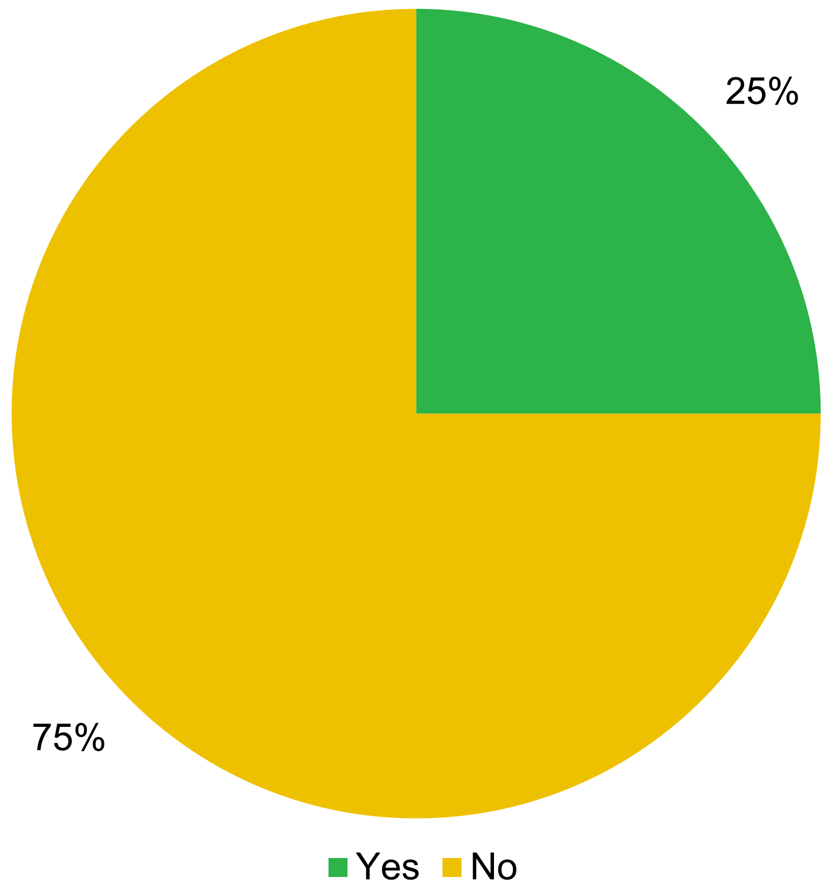

N=56 respondents from 32 countries

If there is a preferred structure for asthma reviews, what structure is used?

- Templates are used on electronic systems (short or long) (UK)
- ACT (Taiwan, Spain, Sweden)
- Recommendation from the Portuguese Primary Care Respiratory Group (Portugal)
- Asthma questionnaire (Egypt)
- Quality and outcomes framework review (UK)
- Assessing patient's symptoms (Sweden)
- Checking exacerbations, treatment adherence, inhaler technique, comorbidities and patient's satisfaction (Spain)
- Structured asthma clinic (Uganda)
- Team review (USA)
- National guidelines for non-communicable diseases (Bangladesh)
- SIMPLES (Romania)

ACT, Asthma Control Test

Are people with asthma referred in a timely manner to secondary healthcare professionals when their symptom control is poor and cannot be managed in primary care?

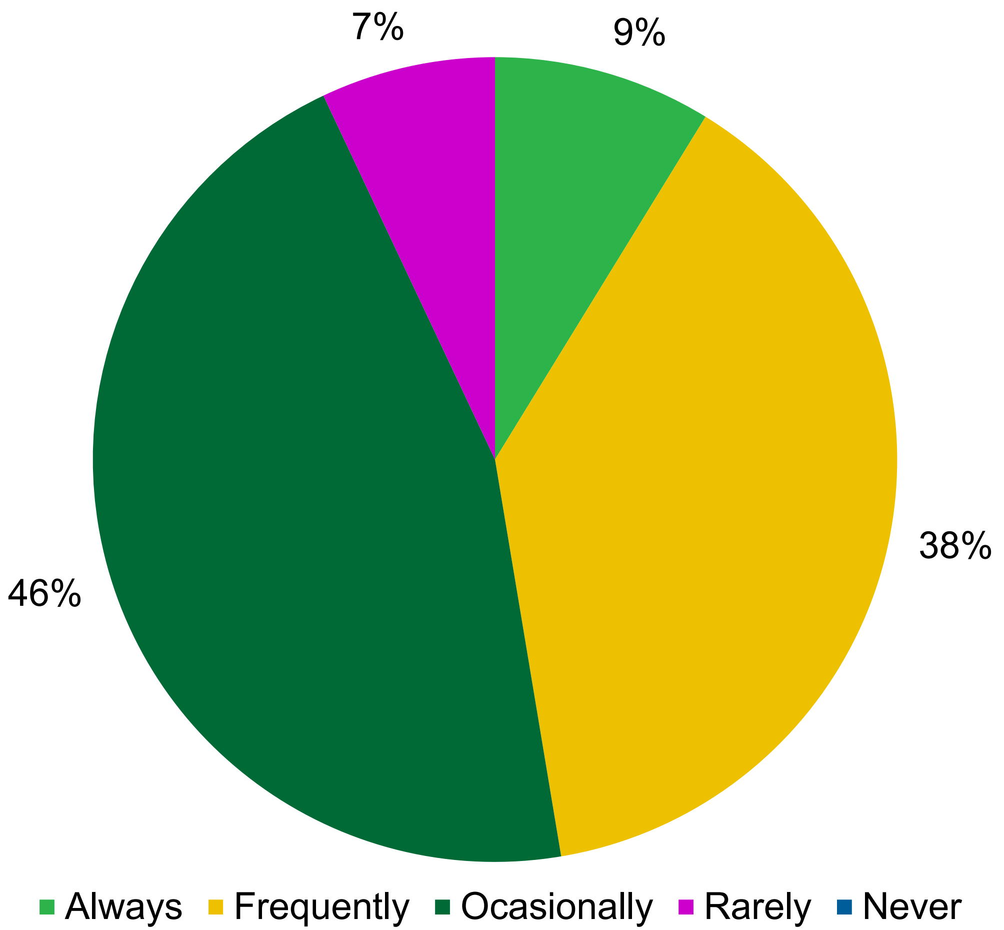

N=57 respondents from 33 countries

If people with asthma are referred in a timely manner, what are the processes in place to enable that?

- Recognition of poor asthma control may not be there (patient and clinician); this is improving since system searches can be done to identify patients at high risk and invited in for an urgent review (UK)
- Electronic referral (Taiwan, UK)
- Referral software ‘Alert P1’ is used at national level in the healthcare system (Portugal)
- Referral system to specialist (Egypt, Spain, Colombia, Malaysia, Mexico, Greece, South Africa,[a] Sweden, Romania, Uganda, USA, Singapore, Thailand, Spain, North Macedonia, Slovenia, Dominican Republic)
- Patients can access a specialist directly (Egypt, Vietnam, Bangladesh)
- Addressing patients with a letter from their primary care physician (Tunisia)
- Specialised asthma clinics (UK)
- Arrangements between HCPs (Greece)
- Access to private system (but not affordable to everyone) (Australia)

[a]But often specialists are fully booked and patients have to travel to appointments
HCP, healthcare professional

In your country, is there a local/national guideline available for asthma-treating physicians?

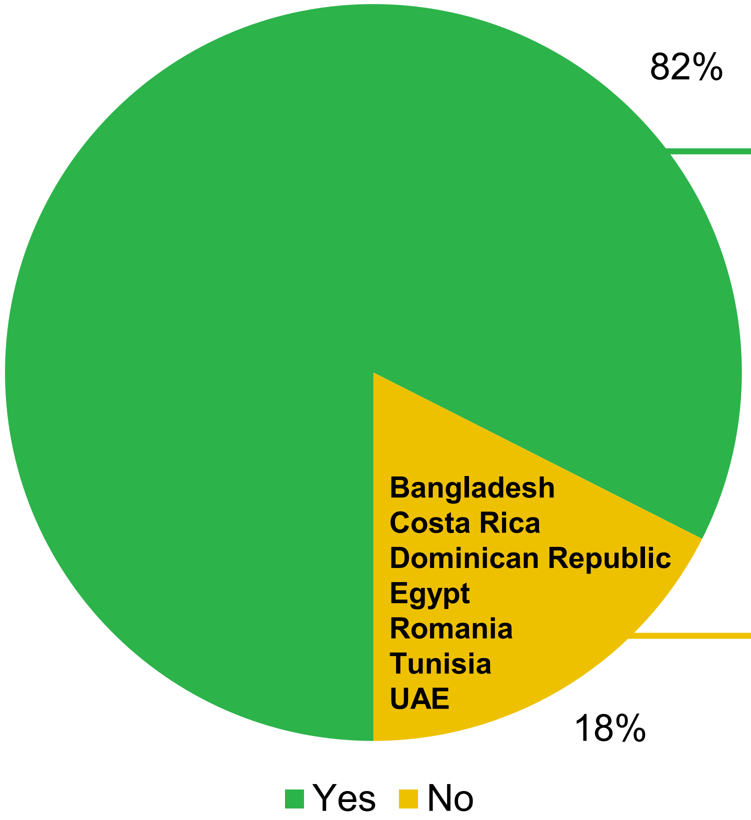

If there is a guideline available, and were primary care practitioners involved in producing this guideline? (n=47)

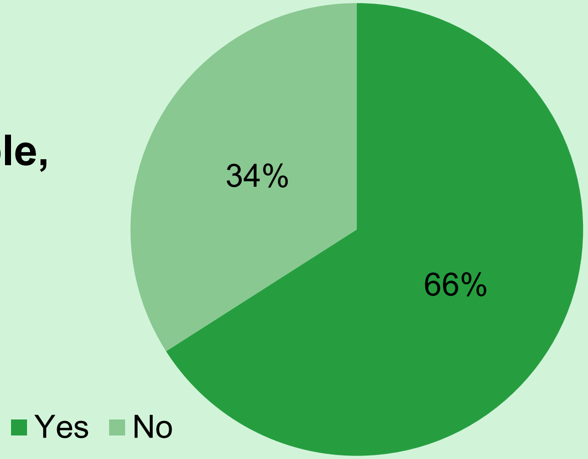

If there is no guideline available, what guideline(s) do asthma-treating physicians follow? (n=10)

GINA

N=57 respondents from 33 countries, unless otherwise stated
GINA, Global Initiative for Asthma

To what extent do you believe this local/national guideline is implemented in clinical practice? (n=46)

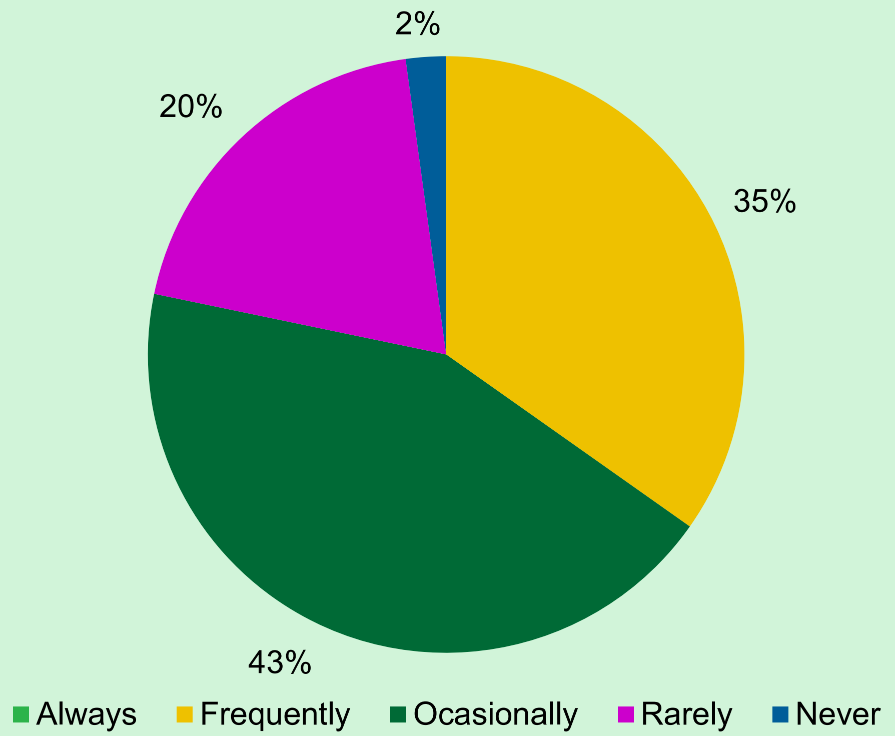

Are there national policies to support the implementation of the local/national guideline in clinical practice? (n=50)

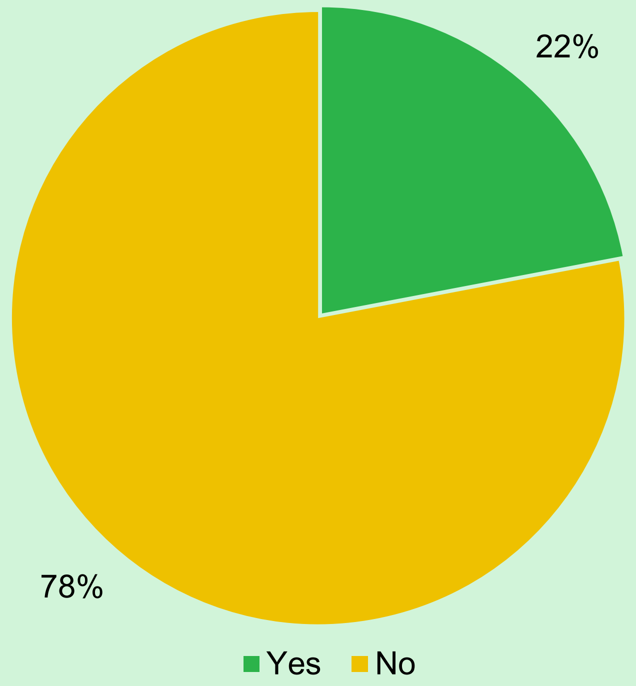

What are the challenges to implementing this guideline in clinical practice?

- Lack of time (UK, Portugal, Spain, Sweden, Greece, Pakistan)
- Lack of workforce/resources (UK, Greece, Malaysia, North Macedonia, Pakistan, Brazil, South Africa)
- Prioritisation (UK)
- Lack of skill set (UK)
- Lack of a structured software for electronic prescription (Portugal)
- Financial aspects (Argentina, South Adrica)
- Lack of education/awareness (Australia, Thailand, Mexico, UK, Kenya, Spain, Uganda, USA, North Macedonia, Pakistan, Brazil, India)
- Lack of access in the private healthcare sector (South Africa)
- Interest (Canada)
- Lack of access to spirometry/FeNO (Malaysia, Mexico)
- Lack of asthma care pathway and medication availability (Malaysia, Mexico)
- Outdated guidelines (Australia, UK, Portugal, USA, Singapore, Romania)
- Guidelines are not easy, short and applicable (Australia, Argentina, Mexico)
- Belief in textbook-based medicine (Bangladesh)
- Guideline not specific to primary care (Thailand)
- Not being able to diagnose asthma in primary care and needing a specialist to do so (Slovenia)

FeNO, fractional exhaled nitric oxide

If national policies to support the implementation of the local/national guideline in clinical practice exist, what are them?

- Quality of outcomes and framework (UK)
- Asthma P4P (Taiwan)
- Clinical indicators; quality improvement projects at local or regional level (Portugal)
- Salbutamol is not sold OTC anymore (Spain)
- Invitation to attend events/courses organised by the ministry of health to promote guidelines (Mexico)
- Local initiatives and long-term plan/incentives to reduce SABA over-reliance (UK)
- Restrictions on the number of prescriptions of certain medications per year; making certain medications unavailable OTC (Argentina)
- Restrictions on OTC medications (Spain, Slovenia)
- Targets/incentives (North Macedonia)
- Training of HCPs (North Macedonia)

HCP, healthcare professional; OTC, over-the-counter; P4P, pay-for-performance; SABA, short-acting β_2-receptor agonist

If there are no national policies to support the implementation of the local/national guideline, what policies could be useful?

- Online consultation (Thailand)
- Targets/incentives (Colombia, Canada, Malaysia, Romania, Spain, Portugal)
- Easy access to electronic data (Colombia, Romania, Spain, Kenya, Mexico)
- Cost of preferred medication (South Africa)
- Availability of medicines (Malaysia, Dominican Republic)
- Updates to local guidelines and implementation of guidelines (Malaysia)
- Education (UK, Bangladesh, Mexico, South Africa, Tunisia)
- Restrict OTC medications (Australia, Spain, Kenya, Romania, Uganda, Greece, Turkey)
- Public health policies and integrated health programmes (Argentina, Brazil, India)
- Review of frequency of SABA prescriptions with feedback to the clinician (Spain, USA, Vietnam)
- Increase access to affordable ICS+LABA inhalers (Singapore)
- Capacity-building and resource allocation for primary care (Thailand)
- National guidelines and referral pathways (Bangladesh)
- Including primary care in policy-making and implementation (Pakistan)
- Increase GPs' role in diagnosis, monitoring and prescription (Romania)
- OCS stewardship (Australia)

GP, general practitioner; ICS, inhaled corticosteroids; LABA, long-acting β_2-receptor agonist; OTC, over-the-counter; SABA, short-acting β_2-receptor agonist

To what extent do you believe that the GINA 2023 track 1 recommendations are implemented in clinical practice?

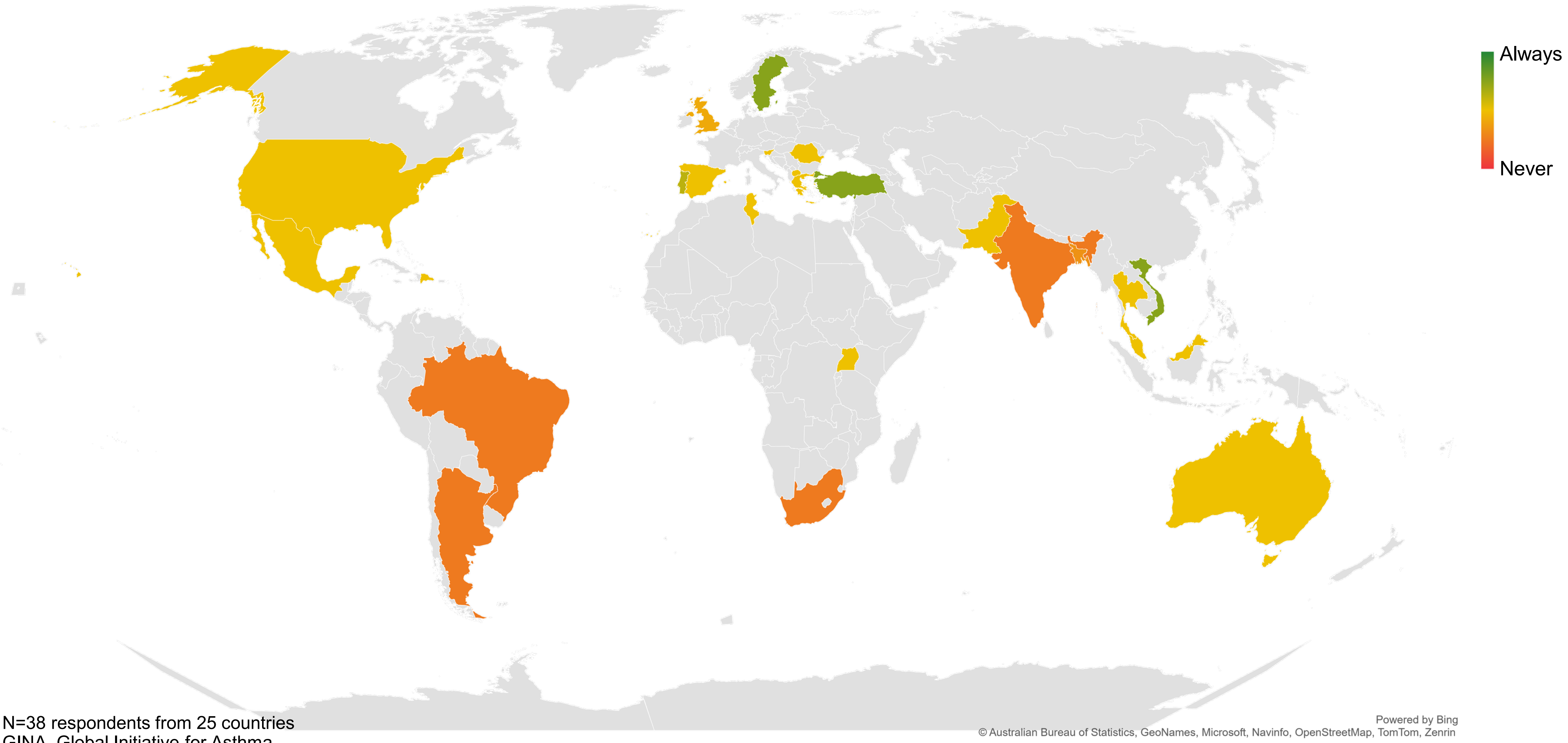

N=38 respondents from 25 countries
GINA, Global Initiative for Asthma

To what extent do you believe that the GINA 2023 track 2 recommendations are implemented in clinical practice?

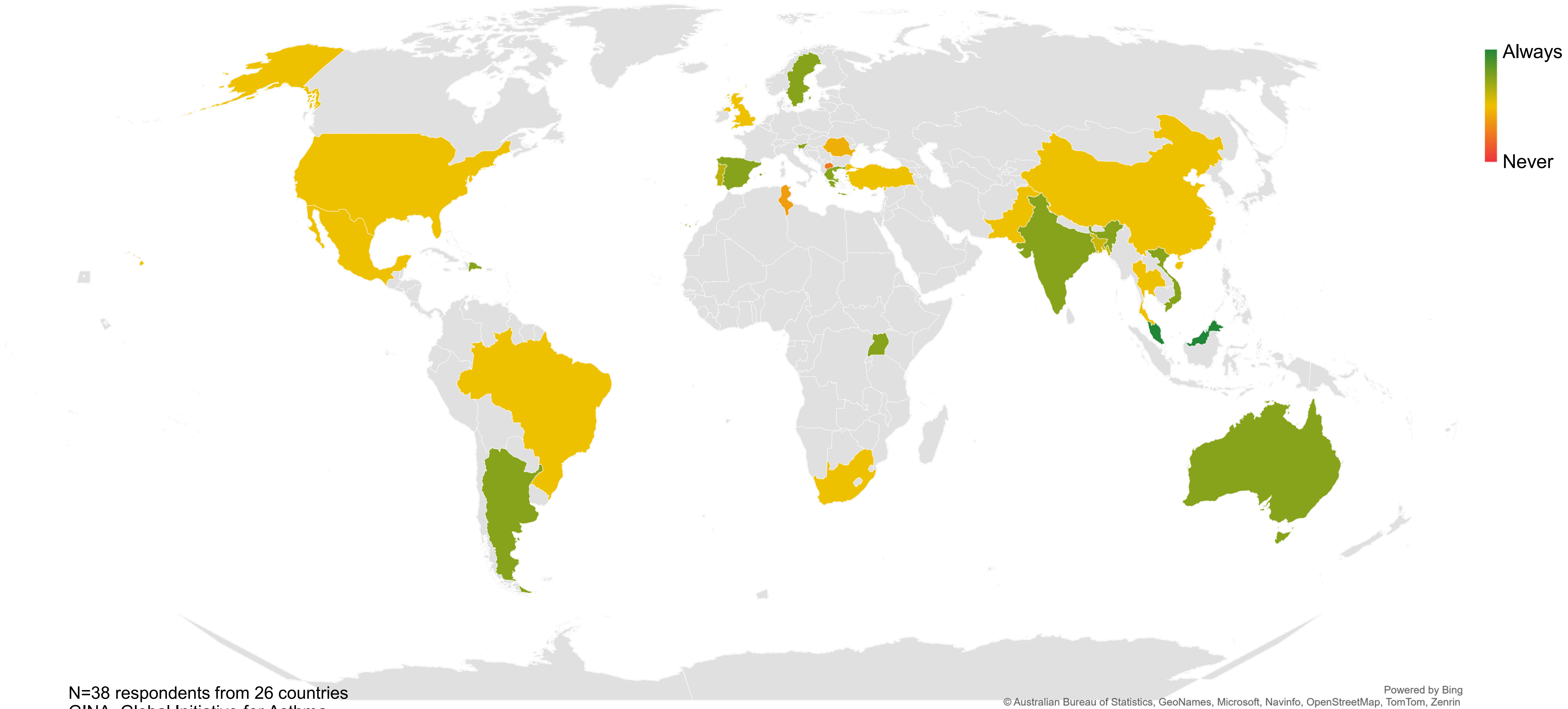

N=38 respondents from 26 countries
GINA, Global Initiative for Asthma

ASTHMA RIGHT CARE

Are there any data to support your view?

- High number of uncontrolled patients (South Africa)
- Some papers published use track 2 (Malaysia)
- ICS-FORM is not available for many primary level attended patients (Mexico)
- Clinical experience (Spain, Romania, Bangladesh, Uganda, Dominican Republic)
- https://www.nhsbsa.nhs.uk/access-our-data-products/epact2/dashboards-and-specifications/respiratory-dashboard (UK)
- Work with this information will be published soon (Argentina)
- Data collection process under way (India)
- Increase of prescriptions of BUD-FORM (Portugal)
- Clinical practice guidelines are similar to track 1 and 2 (Thailand)
- Common practice to use ICS as a reliever in the settings where ICS is available (Thailand)
- Track 2 is more frequently used since the cost of medicines is more accessible (India)

ICS-FORM, inhaled corticosteroids + formoterol; BUD-FORM, budesonide + formoterol

What are the challenges to implementing GINA 2023 track 1 and track 2 recommendations in clinical practice, if any?

Lack of clinicians' awareness/education (Australia, UK, Spain, Mexico, Tunisia, UAE, Kenya, Greece, Sweden, Bangladesh, Uganda, USA, Slovenia, Pakistan, Brazil, India, Romania, South Africa)

Access to medication in primary care (Mexico, Costa Rica, Thailand, North Macedonia, Malaysia, Dominican Republic)

Costs of medication (UK, South Africa, Uganda, Singapore, Bangladesh, Mexico, Tunisia)

Disagreement with GINA recommendations (Spain)

Lack of time (Greece)

Feasibility (e.g. use of pulmonary function regularly, access to spirometry) (Egypt, Portugal)

National health insurance system awareness (Taiwan)

Patients' lack of adherence (Romania, South Africa)

Initiating ICS-FORM vs SABA (Colombia)

Existing guidelines are not aligned with GINA 2023 yet (UK, Canada)

Restriction on prescriptions of SMART/MART in clinics (Malaysia)

Lack of access to specialist care (Romania)

GINA, Global Initiative for Asthma; ICS-FORM, inhaled corticosteroids + formoterol; (S)MART, (Symbicort) maintenance and reliever therapy; SABA, short-acting β_2-receptor agonist
